# The clinical significance of low dose biotin supplements (<300μg/day) in the treatment of patients with hypothyroidism: crucial or overestimated?

**DOI:** 10.1186/s13044-023-00162-8

**Published:** 2023-07-17

**Authors:** Nicholas Angelopoulos, Rodis D. Paparodis, Ioannis Androulakis, Panagiotis Anagnostis, Anastasios Boniakos, Leonidas Duntas, Spyridon N. Karras, Sarantis Livadas

**Affiliations:** 1 Endocrine Unit, Athens Medical Centre, Athens, Greece; 2 Private Practice, 26G Venizelou St, 65302 Kavala, Greece; 3 Center for Diabetes and Endocrine Research, University of Toledo College of Medicine and Life Sciences, Toledo, OH USA; 4 Private Practice, Patras, Greece; 5 Unit of Reproductive Endocrinology, 1St Department of Obstetrics and Gynecology, Medical School, Aristotle University of Thessaloniki, Thessaloniki, Greece; 6 Unit of Endocrinology, Diabetes and Metabolism, Evgenideion Hospital, University of Athens, 11528 Athens, Greece; 7 Laboratory of Biological Chemistry, Medical School, Aristotle University of Thessaloniki, Thessaloniki, Greece

**Keywords:** BCS, Hypothyroidism, TFTs

## Abstract

**Background:**

In the last decade, the combination of the widespread use of streptavidin–biotin technology and biotin–containing supplements (BCS) in the daily clinical practice, have led to numerous reports of erroneous hormone immunoassay results. However, there are no studies assessing the clinical and biochemical significance of that phenomenon, when treating patients with hypothyroidism. Therefore, a prospective study was designed to investigate the potential alterations in the measurement of thyroid hormone concentrations and clinical consequences in patients with hypothyroidism using low -dose BCS containing less than 300 μg/day.

**Methods:**

Fifty-seven patients on thyroxine supplementation, as a result of hypothyroidism and concurrent use of BCS at a dose <300μg/day for 10 to 60 days were prospectively evaluated. Namely, TSH and free T4 (FT4) concentration measurements were performed, during BC supplementation and 10 days post BCS discontinuation and compared to 31 age-matched patients with supplemented hypothyroidism and without BCS.

**Results:**

A statistically significant increase in TSH and decline in FT4 concentrations was observed after BCS discontinuation. However, on clinical grounds, these modifications were minor and led to medication dose adjustment in only 2/57 patients (3.51%) in whom TSH was notably decreased after supplement discontinuation.

**Conclusion:**

Our study suggests that changes in thyroid hormones profiling, due to supplements containing low dose biotin, are of minimal clinical relevance and in most cases don’t occult the need to adjust the thyroxine replacement dose in patients with hypothyroidism. Larger, well-designed trials are required to further evaluate this phenomenon.

## Background

Precision in clinical endocrinology is strongly dependent on the accuracy of hormonal measurements, as hormone quantification is the main determinant of diagnosis, prognosis and treatment selection in endocrine disorders. However, a number of factors may affect the analysis of thyroid hormones, which should be considered to interpret thyroid function tests (TFTs). Biotin is a component of the vitamin B complex used in several immunoassays to detect serum levels of various hormones including the commonly used tests for assessing thyroid function, such as thyrotropin stimulating hormone (TSH) and free thyroxine (FT4). On the other hand, the widespread use of over-the-counter biotin supplements for medical and non-medical purposes is thought to adversely affect the diagnosis of the entire spectrum of functional thyroid disorders [1].

Biotin has a high affinity for streptavidin, and this phenomenon has been used in the design of competitive FT4, total triiodothyronine (TT3), and non-competitive immunometric assays (TSH) [1, 2]. In both types of assays, an excessive amount of biotin intake could result in low signal production, although the result highly depends on the assay design: falsely high concentrations are observed in competitive immunoassays, such as those used to measure FT4 and TT3, and falsely low levels are observed in immunometric “sandwich” assays, such as those used for TSH evaluation [2]. The use of high-dose biotin-containing supplements for non-medical indications has been increased in recent years, along with the use of several vitamin-containing supplements. Since the prevalence of hypothyroidism in the global population is rising [3], and clinicians rely on the frequent measurements of thyroid function to adjust the levothyroxine (LT4) replacement dose, there is potential for clinical mismanagement of these patients based on misleading test results with unclear consequences for patients’ safety [4, 5].

Manufacturers have made assays less vulnerable to biotin interference, however, high-dose biotin therapy resulting in increased serum concentrations, could affect the most sensitive assays that present profound analytical bias. Though some assays, such as troponin T, TSH, and antithyroid antibodies, were found extremely sensitive to the lower concentrations of biotin (15.6 and 31.3 ng/mL), the majority of assays were relatively resistant. In contrast, at biotin concentrations ≥ 500 ng/mL, all assays showed significant interference from biotin with a variable magnitude of interference [6].

In order to address this issue, we designed the present study, aiming to assess the effects of biotin interference in thyroid function laboratory measurements in patients treated with LT4 for hypothyroidism, during their usual clinical and laboratory assessment with a comparison of different commonly used biotin-containing supplements in doses < 300μg/day. In addition, we assessed the effects of our findings, with regard to patient care clinical decisions regarding thyroxin dose adjustment.

## Methods

### Study population

The present study was designed to explore the relationship between the use of over-the-counter biotin-containing supplements and their potential impact on thyroid hormonal profile in patients with long-standing hypothyroidism. Since the vast majority of multivitamins available in our country contain biotin doses less or equal to 300 mg /day we enrolled patients receiving supplements with this cutoff of biotin concentration. Therefore, we recruited 57 consecutive hypothyroid patients under BCS for more than 10 days and less than two months, who were examined in an outpatient basis between January 2022–May 2022. All patients had thyroid hormone measurements within the reference range, during their last annual examination and were on a standard dose of levothyroxine treatment during the study.

The daily biotin dose intake was based on the summary of product characteristics, of their respective over-the-counter (OTC) preparations. Commonly, BSC prescription aimed to promote skin, nails or hair vigor via the intake of multivitamin preparations. Participants, who were not taking their BCS consistently, or who used these supplements for less than 10 days prior to study enrollment, patients taking medications (other than thyroxine and biotin) that might influence thyroid hormone measurements, such as supplements containing (iodine,corticosteroids,amiodarone) were excluded. Additionally, pregnant and lactating women, and patients with renal insufficiency (glomerular filtration rate < 60 ml/min/1.73m^2^) and/or with liver function tests abnormalities (aspartate transaminase/alanine transaminase above the reference range, > 40 U/l and 56U/l respectively) were excluded.

In all subjects, morning fasting blood samples were collected after 10-h overnight fasting between 7–8 a.m. on two separate occasions: as a baseline measurement in patients consistently using biotin-containing supplements and 10 to 15 days following biotin withdrawal, while the thyroxine dose was kept unaltered. Control group consisted of 31 age-matched well treated hypothyroid patients without BCS, who are followed up annually in our outpatient clinics, in whom both TSH and FT4 were reassessed 15 days after the initial measurement.

## Methods

Serum aliquots were analyzed for TSH and FT4 quantification in two platforms, chosen based on the use of a streptavidin/biotin system: Roche Cobas 6000 (Roche Diagnostics Ltd, Basel, Switzerland, using the Elecsys TSH and FT4 III immunoassays) and Siemens IMMULITE 2000 (Siemens Healthcare Diagnostics Ltd, Munich, Germany, using Immulite 2000 TSH and FT4 assays). TSH was measured by an electrochemiluminescence immunoassay with a reference range of 0.45‐4.50 mIU/L; FT4 was analyzed by a chemiluminescent immunoassay with a reference range of 0.7 ‐ 1.5 ng/dL. In both TSH and FT4 assays streptavidin/biotin either in the form of coated microparticles (TSH) or coated at the solid phase (FT4) was used for secondary signal amplification system.

### Statistical analysis

The normality of the distribution of our data was assessed using the Kolmogorov–Smirnov test. A paired t-test was used to compare within-group differences in normally distributed parameters (FT4), while intragroup differences in skewed distributed data (TSH) were analyzed by using the Wilcoxon non-parametric test. Moreover, differences between time points were indicated in percentage. The Pearson and Spearman correlation tests were used to investigate potential associations between variables. Statistical Package of Social Sciences 21.0 (SPSS Inc, Chicago, Illinois, USA) was used for the statistical analysis of anthropometric and laboratory chemical data. All values are presented as mean age ± standard error mean (SEM). The null hypothesis was always rejected for values of *p* < 0.05.

## Results

From the initial population of 112 consecutive patients who were screened to participate in the study, while using biotin containing OTC supplements, *n* = 57 (50 women and 7 men, age range 15 to 78 years old, [49.12 ± 2.09 years]) fulfilled the inclusion criteria and were recruited into the study. Body mass index (BMI) 25.2 ± 0.57 kg/m^2^ didn’t change during the study period. The mean dose of thyroxine treatment was 66.85 ± 3.97 μg daily. The cause for thyroxine supplementation was hypothyroidism due to Hashimoto’s thyroiditis (85%) and thyroidectomy (15%). The amount of biotin in the supplements was 91.53 ± 9.25 μg (range 20–300 μg daily) as shown in Table 1.

**Table 1:** Demographic characteristics

	Sex (M/F)	Age, years (15–78)	BMI (Kg/m^2^)	Disease		Thyroxine dose (μg/day)	Biotin dose (μg/day) (range 20–300 μg)
				Hashimoto N/%	Thyroidectomy N/%		
Biotin use *N* = 57	7/50	49.12 ± 2.09	25.2 ± 0.57	49/85%	8/15%	66.85 ± 3.97	91.53 ± 9.25
Controls *N* = 31	3/28	51.17 ± 2.38	24.9 ± 0.81	26/84%	5/16%	63.33 ± 5.37	
*P*	0.998^b^	0.546^a^	0.348	0.762^b^		0.944	

*N* Number of patients, *M* Male, *F* Female, *BMI* Body mass index, P, Mann Whitney test for unpaired data

^a^Unpaired t test for normally distributed values

^b^Fisher's exact test; Data presented as means ± SEM

**Fig. 1:**
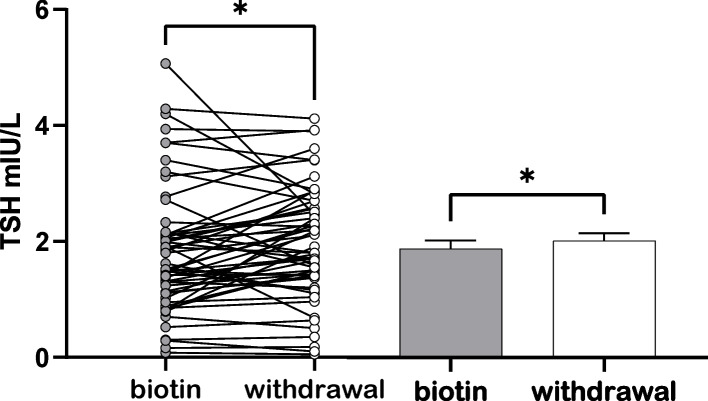
Thyroid-stimulating hormone levels alterations. Thyroid-stimulating hormone (TSH, mIU/L) under biotin containing supplements (gray bar) and 10-15 days after withdrawal (white bar); * *p*<0.05.  Data presented as means± SEM

**Fig. 2:**
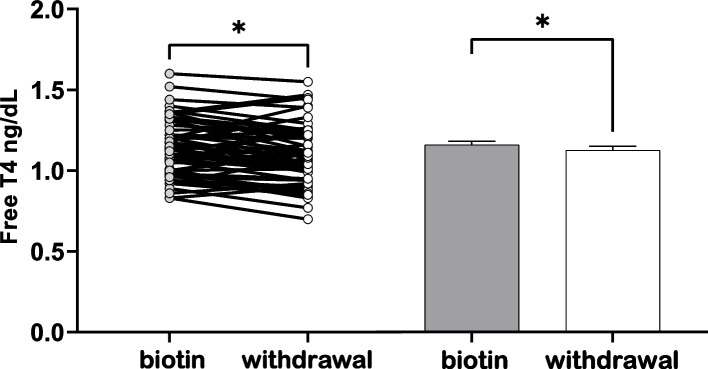
Free thyroxin levels alterations. Free thyroxin levels (FT4, ng/dL) under biotin containing supplements (gray bar) and 10-15 days after withdrawal (white bar); * *p*<0.05. Data presented as means± SEM

After BCS discontinuation, TSH increased significantly compared to that measured prior to BCS withdrawal (1.95 ± 0.12 mUI/L vs. 1.82 ± 0.14, *p* = 0.029) while FT4 levels decreased significantly (1.10 ± 0.02 ng/dL vs. 1.13 ± 0.02 *p* = 0.024) as illustrated in Figs. 1 and 2 and Table 2. Compared with controls, there was a significant decrease in FT4 levels after biotin discontinuation.

**Table 2:** Laboratory findings at baseline (t0) and reassessment (t1)

Parameter	Group	t_0_	t_1_	t_1_- t_0_ (mean, 95% CI)	
				Difference	% Difference
TSH	Treated (*N* = 57)	1.82 ± 0.14	1.95 ± 0.12	0.13( -1.23; 1.5)	16.62 (-75.45; 108.69)
	Controls (*N* = 31)	2.13 ± 0.15	2.18 ± 0.13	-0.05 (-0.78; 0.68)	-2.22(-35.5; 31.07)
	*P* value (T vs C)	0.091	0.290		
FT4	Treated	1.13 ± 0.02	1.10 ± 0.02	-0.03 (-0.24; 0.17)	-2.64 (-21.05; 15.78)
	Controls	1.17 ± 0.02	1.19 ± 0.02	0.02(-0.14; 0.18)	1.55(-11.77; 14.86)
	*P* value (T vs C)	0.216	0.024		

*TSH* Thyroid-stimulating hormone concentrations in mIU/L, *FT4* Free thyroxin levels in ng/dL, *T-group* Treated-group, *C-group* Control-group, *t0* Basal measurement, *t1* Second measurement, *N* Number of participants, *%* Percentage of difference

P, Mann–Whitney U test; Data presented as means ± SEM

In two patients changes in TSH levels (from 4.21 to 2.82 and 5.06 to 2.46 mUI/L) post biotin withdrawal was considered clinically important (led to dose modification) and, in discordance with the pool data statistics, no modification of FT4 values was noticed in these two subjects.

Regarding the impact of biotin dose on thyroid hormone levels, no statistically significant correlation was detected between the biotin dose ingested in either TSH (*r*^2^ = -0.09, *p* = 0.53) or FT4 levels (*r*^2^ = 0.08, *p* = 0.52). Present study findings were further analyzed based on biotin dose used and available data are depicted in Table 3. Significant increase in TSH levels was revealed only in the subgroup of patients that were taking BCS in doses > 100 μg daily.

**Table 3:** Pertinent data of studied subjects’ characteristics stratified according to biotin dose

Biotin dose	TSH Biotin	TSH Withdrawal	*P*	FT4 Biotin	FT4 Withdrawal	*P*
Total (*N* = 57)	1.82 ± 0.14	1.95 ± 0.12	0.029^*^	1.13 ± 0.02	1.10 ± 0.02	0.024
≤ 100 μg/day (*N* = 40)	2.13 ± 0.28	2.10 ± 0.19	0.253	1.13 ± 0.03	1.11 ± 0.02	0.160
> 100–300 μg/day(*N* = 17)	1.62 ± 0.17	1.89 ± 0.20	< 0.05	1.13 ± 0.04	1.08 ± 0.04	0.07

*TSH* Thyroid-stimulating hormone concentrations in mIU/L, *FT4* Free thyroxin levels in ng/dL, *N* Number of patients, P, Wilcoxon nonparametric test

^*^ paired t-test; Data presented as means ± SEM

## Discussion

Biotin recommended intake of 30 μg/day is easily obtained from a balanced diet, and real biotin deficiency seems to be a very rare condition in the modern world. Biotin is present in some popular dietary supplements, alone or as a component in multi-vitamin formulas. It is advertised as a remedy for losing hair and for the fragility of hair and nails (< 20 mg/d) [4].

Automated immunoassays used to evaluate thyroid function are vulnerable to different types of interference that can affect clinical decisions [2]. However, in recent years, the widespread use of streptavidin–biotin technology, and the common use of biotin-containing supplements, has led to numerous reports of erroneous results of hormone- and non-hormone parameters measured by immunoassays [5–8]. In the vast majority of the studies previously reported, the amount of biotin ingested was in the range of moderate to high doses, and biotin (> 10 mg/day) interference resulted in either falsely high or low values.

Interestingly enough, interference has not been limited to thyroid tests and has the potential to affect a wide range of analytes, such as thyreoglobulin (Tg), luteinizing hormone (LH), follicle stimulating hormone(FSH) e.t.c [2, 6]. Namely, Biotin can inhibit immune complex separation, leading to analytical errors in some patient’s labwork; this may be encountered in biotin supplementation or in the presence of anti-streptavidin antibody [2]. In these cases, the interference may induce both false positive and false negative results, and simulate a seemingly coherent hormonal profile [5, 7]. This bidirectional behavior with opposing effects may mimic the biochemical diagnosis of hyperthyroidism with falsely elevated concentrations of FT4, free T3 (FT3), anti-TSH receptor Ab, and falsely lowered TSH concentration [6, 7]. Conversely, several endocrine disorders, including primary hypothyroidism, may be misdiagnosed because of biotin distortion of the assays decreasing the elevated TSH levels, primarily in patients with chronic kidney disease or biotinidase deficiency [2, 8–10]. The reformulation of immunoassays for biotin-resistant methods is in progress, but biotin-susceptible methods remain in widespread use throughout the world [11].

A topic remaining incompletely understood is the duration of the washout period needed in subjects consuming BCS, for the accurate evaluation of either TSH or FT4 levels. Based on available literature data the washout periods in several studies ranged from a few hours to 8 days [12, 13]. Given the variability in these results, it has been impossible to provide a consensus recommendation on that issue [8, 14]. The impact of biotin supplements on thyroid hormone levels can differ depending on the amount and length of time biotin has been taken [12, 13]. Therefore we focused on patients where BCS was received from at least 10 days to two months. One the other hand, once biotin interference is suspected to last from several hours to several days, we consider that the reassessment of thyroid hormones 15 days after supplements withdrawal is probably a sufficient wash out period to provide us with solid data.

The main goal of the present clinical study was to investigate whether various biotin supplements alter thyroid hormone profiles in hypothyroid patients and to what extent that “biochemical fault” might lead clinicians to unnecessary changes in the amount of prescribed thyroxine dose to their patients. We investigated potential alterations in the measurement of TSH and FT4 seen in real world practice, since these two parameters are used commonly as part of the assessment of patients with hypothyroidism both in endocrine, but also in non-specialty clinics, such as Internal Medicine and General Practice.

Studies have tested various doses of biotin, which were given to volunteers and demonstrated that the extent of analytical error is less remarkable as far as endocrine assays are concerned. The dose applied was approximately 100 times higher than the required daily dose (30–100 µg), and corresponded well to the amount of biotin found in commercial dietary supplements [15].

Using a moderately high intake such as 10 mg daily [15], it may lead to inaccurate results. The major determinants of such errors for susceptible immunoassays are the biotin dose; time elapsed since last intake, and kidney function [14–17]. Various factors may contribute to the artifactually falsely high or low results, including the degree of blood biotin elevation based in terms of the amount of biotin ingested, the time interval from biotin ingestion to blood specimen collection, the biotin interference threshold, and the patient's own relative biotin metabolism.

Although supra-nutritional amounts of biotin are often taken over the counter for hair, skin and nail benefits in the thousands of micrograms many multivitamin supplements contain biotin in smaller amounts, ranging from 20 μg to 300 μg and this probably explains the lack of any effect in thyroid hormone levels, observed in some studies. Recently, the high-dose biotin (100 mg to 300 mg/day, which is 10,000 times the standard dietary reference intake) has been accepted as a valid treatment strategy in patients with progressive multiple sclerosis [18], potentially threatening an overflow of abnormal TFTs in treated patients. Therefore, cautious interpretation of measured thyroid parameters should be exerted in such circumstances, or a biotin insensitive assay should be used.

The discrepancy between a clinical exam which is not indicative of thyroid dysfunction and markedly abnormal thyroid function tests should lead to a search for biotin intake, which can interfere with thyroid function tests. Additionally, most patients fail to mention taking this supplement to their physician, because it is not seen as a “real medication.” Both clinicians and patient awareness of this issue are necessary in high doses of biotin intake supplements. However, in daily practice, the impact of most multivitams (containing lower than 300 μg of biotin) seems to be irrelevant in terms of clinical decision and no further reassessment is needed.

We understand that the present study has two major limitations: First is the lack of biotin concentrations measurement in our cohort. Previous studies however have evaluated the biotin concentration in patient samples from different countries. A study quantifying biotin in plasma samples from emergency department patients in the USA showed that 7.4% of samples had a biotin concentration at or above 10 ng/mL (considering a low threshold, above which interference will occur for the most sensitive immunoassays) [19]. In another study from Australia, 0.8% (4/490) of subjects sampled presented with a biotin concentration above 10 ng/mL, whereas in the Netherlands, this percentage was estimated to be 0.2% [20, 21]. Secondly, the absence of patients receiving larger doses of biotin that could lead to major alterations in TFTs as recently mentioned [22]. The biotin interference threshold depends on the test and the platform used [23]. It is conceivable that the interference can become progressively more significant with higher biotin levels or less significant with biotin levels below the threshold. Since the process of biotin measurement is cumbersome and costly, we aimed straight to the clinical impact of supplement ingestion in daily clinical practice. For the same reasons, our protocol is mimicking the “real world” tactic where only TSH and/or FT4 are used to assess thyroid status in patients with chronic hypothyroidism receiving usual over the counter multivitamins.

Alterations observed in our study in both ΤSH and FT4 levels, (although reached statistical significance) were modest and cannot be solely attributed to analytical interference of biotin with the applied assays. Of note, in the only two patients where clinical decision might be misleaded, TSH was decreased after supplement withdrawal, opposite than expected. Differences between functional sensitivities can be found among laboratories using the same automated system even in the same laboratory. These discrepancies can be attributed to the way sera pools are prepared, the periodicity of measuring, and even to differences among automated systems that are theoretically the same [24]; whether recommended calibration prosses is performed regularly remains also questionable. Among the important criteria that influence desirable analytical performance is the biological variation of a given parameter [25]. Seasonal variations in thyroid hormones levels have been documented previously, mainly assigned to alterations in the central sensitivity to thyroid hormones (increased in summer and decreased in winter) [26]. Hence, the statistical difference in this study should not be interpreted uncritically as clinical significance. Unfortunately, a knowledge gap amongst physicians regarding the risks and benefits of biotin supplementation still exists. A recent study reported that almost half of physicians did not advise biotin cessation prior to laboratory testing, implying knowledge of biotin interference may not translate into change in practice [27].

## Conclusion

In conclusion, thyroid function testing in the setting of concurrent biotin containing supplements intake should be cautiously interpreted. Based on our findings we suggest that in patients with long standing hypothyroidism treated with LT4, no major changes in the treatment strategy are needed when supplements used containing biotin in concentrations < 100 μg/day. However, larger studies are needed to further exploit this issue.

## Data Availability

The data that support the findings of this study are available on request from corresponding author.

## Abbreviations

BCS: Biotin–containing supplements
TSH: Thyroid‑stimulating hormone
FT4: Free thyroxin
TFTs: Thyroid function tests
TT3: Total triiodothyronine
LT4: Levothyroxine
OTC: Over-the-counter
